# A natural history study of Chinese individuals with Duchenne muscular dystrophy: Results from 2 years of follow-up and beyond

**DOI:** 10.1371/journal.pone.0345023

**Published:** 2026-04-02

**Authors:** Xihua Li, Xinying Yang, Xingzhi Chang, Zhiqiang Wang, Siqi Hong, Wenhua Zhu, Qi Shen, Yangmei Zhou, Shuiqing Zhu, Ying Xu Gao, Caiping Jia

**Affiliations:** 1 Children’s Hospital of Fudan University, Fudan, China; 2 Department of Neurology, Beijing Children‘s Hospital, Capital Medical University, National Center for Children’s Health, Beijing, China; 3 Peking University First Hospital, Peking, China; 4 The First Affiliated Hospital Of Fujian Medical University, Fujian, China; 5 Children’s Hospital of Chongqing Medical University, Chongqing, China; 6 Department of Neurology, Huashan Hospital, Fudan University, China; 7 Pfizer Inc, New York, United States of America; 8 Pfizer, Shanghai, China; 9 Pfizer, Beijing, China; Fondazione Policlinico Universitario Gemelli IRCCS, ITALY

## Abstract

**Background and Objectives:**

Natural history studies in Duchenne muscular dystrophy (DMD) can help to elucidate the trajectory of the disease, understand the importance of clinical milestones, and identify endpoints for clinical trials. We present data from a natural history study of DMD in Chinese individuals with a follow-up of up to 30 months.

**Methods:**

This was a multicenter, prospective, single-cohort study in Chinese boys of any age with a confirmed diagnosis of DMD. Participants were allocated into one of three groups: Group 1 (ambulatory, < 6 years old); Group 2 (ambulatory, ≥ 6 years old); and Group 3 (non-ambulatory, any age). Endpoints included time to life-altering clinical milestones, North Star Ambulatory Assessment (NSAA; Groups 1 and 2), Performance of Upper Limb 2.0 (PUL2.0; Groups 2 and 3), pulmonary function (Groups 2 and 3), left ventricular ejection fraction (LVEF; Groups 2 and 3), and quality of life endpoints.

**Results:**

312 participants were enrolled (Group 1, n = 99; Group 2, n = 177; Group 3, n = 36). Mean (SD) age at baseline in the total study population was 7.9 (3.4) years. Median (95% confidence interval) age at failure to walk in the total study population was 13.1 (12.5–14.1) years. Up to 30 months, NSAA total score deteriorated in Group 2 with no decline in Group 1. PUL 2.0 score declined in both Groups 2 and 3. Pulmonary function decreased in Groups 2 and 3 but to a greater extent in Group 3. Changes in LVEF were minimal. Quality of life deteriorated over time, particularly in Group 3.

**Discussion:**

These data extend the 1-year findings from the same study and demonstrate the progression of DMD in Chinese individuals of differing age and ambulatory status.

**Registration information:**

ClinicalTrials.gov identifier: NCT03760029.

## Introduction

Duchenne muscular dystrophy (DMD) is an X-linked, recessive, severe muscle-wasting disease caused by variants of the *DMD* gene, resulting in a reduction or the complete absence of the muscle isoform of dystrophin [1]. Progressive muscle degeneration affects ambulation and upper limb function, and eventually results in musculoskeletal deformities, respiratory and cardiac complications, and premature death [1]. Systemic glucocorticoid therapy is a mainstay of treatment, delaying the time to key disease progression outcomes and extending lifespan [2]. In recent years, more treatment options have become available [3–6]. This includes vamorolone a dissociative agonist of the glucocorticoid receptor that has recently been approved in multiple countries including China [7,8]. Furthermore, gene therapies are in development that introduce a truncated form of dystrophin to skeletal muscle with the aim of restoring dystrophin function [9,10], such as delandistrogene moxeparvovec, recently approved in the United States [11]. However, an unmet need exists to develop new treatment options that can ameliorate symptoms or modify the disease course [12,13].

Given the long-term nature and heterogeneity of DMD natural history studies play an important role in helping to more fully elucidate the trajectory of DMD [14,15], understand the importance of clinical milestones [16], identify and develop potential biomarkers or endpoints for clinical trials [17,18], or be used as external control groups for drug development and evaluation [19]. Few natural history studies have been conducted in Chinese populations [20–24], therefore the time course of DMD in these individuals is not fully understood. Establishing the natural history of DMD in Chinese populations may also be important given the increasing likelihood of global clinical trials incorporating different geographic populations. Study C3391004 is a large, prospective, single-cohort, natural history study of Chinese individuals with DMD receiving standard-of-care treatment with a follow-up period of at least 24 months. Data at 12-months follow-up have been reported [25]. By the 12-month cutoff, the time to disease progression milestones were similar to previous studies, and there was a trend toward a decline in motor and pulmonary function [25]. Here, we extend the results from study C3391004 out to 2 years and beyond to evaluate the longer-term progression of DMD in this cohort.

## Materials and methods

### Study design

C3391004 is a multicenter, prospective, single-cohort, natural history study of DMD in male Chinese patients. The study has been described in detail elsewhere [25]. Chinese males of any age with a confirmed diagnosis of DMD in their medical history and by genetic testing were included. Standard of care treatment was defined as participants ≥4 years old receiving glucocorticoids as prescribed by their physician for a minimum of 6 months, including on a stable regimen for ≥3 months. Intermittent dosing was not permitted. Exclusion criteria included any injury at baseline that may have impacted functional testing, the presence or history of other musculoskeletal or neurologic disease or somatic disorder not related to DMD, and participation in an investigational study or studies within 90 days prior to study entry and/or during the study itself. Upon entry into the study, participants were allocated into one of three groups: Group 1, ambulatory participants <6 years old at screening; Group 2, ambulatory participants ≥6 years old at screening; and Group 3: non-ambulatory participants of any age at screening. Each participant was observed for at least 24 months. Participants who completed the Month 24 visit at least 6 months prior to study completion were asked to complete an additional visit at Month 30.

### Primary endpoints

Endpoints assessing the time to life-altering clinical milestones due to DMD progression were age at failure to walk (inability to perform 10-m walk run [10MWR] test as part of the North Star Ambulatory Assessment [NSAA]), age at failure to stand, and age at failure to self-feed. Motor function, muscle strength, and range of motion assessments were carried out at baseline and 6, 12, 18, 24, and 30 months. NSAA total score (in ambulatory participants ≥3 years old) and Performance of Upper Limb 2.0 (PUL2.0) total score (in participants ≥10 years old) were used to assess motor function. NSAA is a 17-item assessment that grades performance of various functional skills using the following scale: 0 (unable to perform), 1 (completes independently but with modification), or 2 (completes without compensation). Scores range from 0–34 with higher scores indicating better function. PUL2.0 is a 22-item scale designed to capture motor performance of the upper limb and uses the same scale as NSAA. Scores range from 0–42 with higher scores indicating a higher level of function. In addition, rise from floor velocity (RFF) and 10MWR velocity were captured as part of the NSAA assessment. Muscle strength was assessed for bilateral knee extension, elbow flexion, elbow extension, and shoulder abduction in participants ≥5 years old and quantified by means of handheld myometry. Range of motion was evaluated in bilateral ankles and elbows. Pulmonary assessments were conducted in participants ≥6 years old at baseline and Months 12, 24, and 30. Assessments included percent predicted forced vital capacity (%pFVC), percent predicted forced expiratory volume in 1 second (%pFEV1), maximum inspiratory pressure (MIP), maximum expiratory pressure (MEP), and peak cough flow. Left ventricular ejection fraction (LVEF) measured by echocardiogram was used to evaluate cardiac function at baseline and Months 12, 24, and 30 in participants ≥6 years old. Intellectual ability and cognitive function were evaluated using the Wechsler Intelligence Scale for Children (WISC) at baseline and Month 24 in ambulatory participants ≥6 to ≤16 years old. Higher scores indicate higher cognitive function.

### Secondary endpoints

To characterize the prevalence of DMD mutation types, the proportion of participants with exon deletion, exon duplication, point mutations, small insertion, small deletion, or other types of mutation were determined at baseline. The proportion of each affected exon by mutation type was also captured. The functional health status of participants was assessed using the pediatric parent report, adolescent parent report, and adolescent self-report versions of the Pediatric Outcomes Data Collection Instrument (PODCI). Scores for Global Functioning Scale and each subscale were captured for each version at baseline and Months 6, 12, 18, 24, and 30. Data are reported at baseline and for Months 12, 24, and 30. PODCI scores for each subscale and the Global Functioning scale range from 0–100, with lower scores indication poorer functional health status. Participant’s current health was evaluated at baseline and Months 12, 24, and 30 using the EuroQoL 5 Dimension Youth (EQ-5D-Y) and EuroQoL 5 Dimension 3 Level (EQ-5D-3L). EQ-5D-Y was evaluated in participants <16 years old and EQ-5D-3L in participants ≥16 years old. The EQ-5D-3L consists of 5 dimension (mobility, self-care, usual activity, pain/discomfort, and anxiety/depression) with three levels of severity (no problems, some or moderate problems, extreme problems) within each dimension calculated to form a single index value. EQ-5D-3L also consists of a visual analogue scale (VAS) ranging from 0–100, with higher scores indicating better health. The EQ-5D-Y is a child friendly version of the instrument for use in children and adolescents. Finally, healthcare utilization was evaluated at baseline and Months 12, 24, and 30 using the Work Productivity and Activity Impairment Questionnaire adapted for Caregivers (WPAI:CG). The WPAI-CG consists of four scores (absenteeism, presenteeism, work productivity loss, and activity impairment), each scored as a percentage from 0–100% with higher scores indicating greater impairment and less productivity.

### Standard protocol approvals, registration, and patient consents

This study was conducted in accordance with the protocol, legal, and regulatory requirements and the general principles set out in the International Ethical Guidelines for Biomedical Research Involving Human Subjects (Council for International Organizations of Medical Sciences 2002), International Council for Harmonisation Guideline for Good Clinical Practice, and the Declaration of Helsinki. All participants, or their legally acceptable representative, parent(s), or legal guardian provided written informed consent prior to entry into the study. The study protocol, protocol amendments, informed consent documents, and other relevant documents were approved by the Institutional Review Board/Ethics Committee at each participating center. The study is registered at ClinicalTrials.gov (NCT03760029). The study started on July 24, 2019, and ended on March 21, 2023. The start and end dates of the recruitment period were July 24, 2019, and March 31, 2021, respectively.

### Statistical analysis

The data cutoff for this analysis was April 12, 2023. For continuous endpoints, data were analyzed with descriptive statistics using sample size, mean, and SD. For categorical endpoints, data were analyzed with descriptive statistics using count and percentage. For time-to-event endpoints, median event-free time and survival probabilities were estimated using the Kaplan–Meier method. For subjects with an NSAA Equivalent Activity score of 0 on the item of RFF, RFF velocity was set to 0. For subjects with a missing score, RFF velocity was set to missing. For 10MWR, subjects who could not perform the activity, i.e., Method (Quality) Run/Walk Grade with detail was 1 (unable to walk independently) or 2 (unable to walk independently but can walk with knee-ankle-foot orthosis or support from a person), then 10MWR velocity was set to 0. For subjects with a missing score, 10MWR velocity was set to missing.

## Results

### Participants and characteristics at baseline

Overall, 312 subjects were enrolled in the study and comprised the full analysis set [25]. Ninety-nine (31.7%), 177 (56.7%), and 36 (11.5%) participants were in Groups 1, 2, and 3, respectively. Participant disposition at the end of the study is shown in S1 Fig. In all, 299 (92.9%) participants completed the study and rates of discontinuation were similar across groups. Baseline demographic and participant characteristics are shown in Table 1.

**Table 1:** Baseline demographic and clinical characteristics.

Characteristic	Ambulatory			Nonambulatory	
	Group 1: age <6 y (n=99)	Group 2: age ≥6 y (n=177)	Total (n=276)	Group 3: any age (n=36)	Total (N=312)
Age, mean (SD), y	4.0 (1.2)	9.0 (2.0)	7.2 (3.0)	12.7 (2.7)	7.9 (3.4)
Weight, mean (SD), kg	16.5 (3.1)	28.4 (8.0)	24.1 (8.8)	44.4 (11.7)	26.5 (11.2)
Racial designation, n (%)					
Han Chinese	94 (94.9)	173 (97.7)	267 (96.7)	36 (100)	303 (97.1)
Non-Han Chinese	5 (5.1)	4 (2.3)	9 (3.3)	0	9 (2.9)
Current residence, n (%)					
Urban	77 (77.8)	107 (60.5)	184 (66.7)	20 (55.6)	204 (65.4)
Rural	22 (22.2)	70 (39.5)	92 (33.3)	16 (44.4)	108 (34.6)
Age at DMD diagnosis, mean (SD), y	2.7 (1.2)	5.4 (2.3)	4.4 (2.4)	6.2 (2.0)	4.6 (2.4)
Prior glucocorticoid use					
Yes, n (%)	46 (46.5)	177 (100)	223 (80.8)	36 (100)	259 (83.0)
Duration of use, mean (SD), mo	11.4 (5.9)	30.3 (20.9)	26.4 (20.3)	51.8 (36.6)	29.9 (24.8)
Age at glucocorticoid initiation, mean (SD), y	4.2 (0.5)	6.5 (1.9)	5.8 (1.9)	8.1 (3.2)	6.1 (2.2)
Daily glucocorticoid dose					
Deflazacort, n (%)	6 (6.1)	22 (12.4)	28 (10.1)	2 (5.6)	30 (9.6)
Mean (SD), mg/kg	0.78 (0.09)	0.83 (0.12)	0.82 (0.11)	0.70 (0.16)	0.81 (0.12)
Methylprednisolone, n (%)	0	7 (4.0)	7 (2.5)	1 (2.8)	8 (2.6)
Mean (SD), mg/kg	–	0.48 (0.08)	0.48 (0.08)	0.53 (-)	0.49 (0.07)
Prednisolone, n (%)	7 (7.1)	5 (2.8)	12 (4.3)	0	12 (3.8)
Mean (SD), mg/kg	0.69 (0.05)	0.60 (0.10)	0.65 (0.09)	–	0.65 (0.09)
Prednisone, n (%)	33 (33.3)	143 (80.8)	176 (63.8)	33 (91.7)	209 (67.0)
Mean (SD), mg/kg	0.62 (0.09)	0.60 (0.26)	0.60 (0.24)	0.41 (0.13)	0.57 (0.24)

Participants who were ≥4 years of age must have been receiving glucocorticoid therapy. Duration of glucocorticoid use was calculated as (end date – first date) +1. End date was the last date of glucocorticoid use or enrollment date, whichever was earlier. Daily dose of glucocorticoid in mg/kg was calculated from the total daily dose and baseline weight. DMD, Duchenne muscular dystrophy.

**Fig 1:**
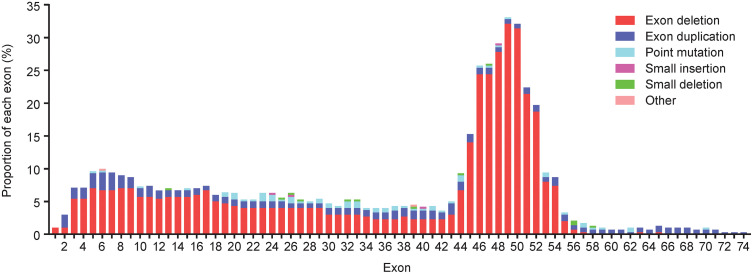
Proportion of each affected exon by mutation type. The denominator to calculate proportion was the number of participants in the overall study population with affected exon.

The mean (SD) age was 4.0 (1.2) years in Group 1, 9.0 (2.0) years in Group 2, 12.7 (2.7) years in Group 3, and 7.9 (3.4) years in the total study population. Patients were enrolled from both urban and rural locations, and the racial designation (Han or non-Han) was approximately representative of the Chinese population. In all, 46.5%, 100%, 100%, and 83.0% of participants had prior glucocorticoid therapy in Groups 1, 2, 3, and overall, respectively, with a respective mean (SD) age at initiation of 4.2 (0.5), 6.5 (1.9), 8.1 (3.2), and 6.1 (2.2) years. These high levels of glucocorticoid use reflect the study protocol where participants ≥4 years old must have received glucocorticoids for a minimum of 6 months, including a stable regimen for ≥3 months. Prednisone was the most commonly used glucocorticoid (33.3%, 80.8%, 91.7%, and 67.0% in Groups 1, 2, 3, and total study population, respectively). The most common DMD mutation was exon deletion in 64.6%, 71.8%, 50.0%, and 67.0% of participants in Groups 1, 2, 3, and total study population, respectively (Table 2). The proportion of each affected exon by mutation type is shown in Fig 1.

**Table 2:** *DMD* mutations.

Mutation types and affected exons	Ambulatory		Nonambulatory	Total (N = 312)
	Group 1: age < 6 y (n = 99)	Group 2: age ≥ 6 y (n = 177)	Group 3: any age (n = 36)	
Abnormality in dystrophin gene, n (%)				
Exon deletion	64 (64.6)	127 (71.8)	18 (50.0)	209 (67.0)
Exon duplication	10 (10.1)	21 (11.9)	6 (16.7)	37 (11.9)
Point mutation	18 (18.2)	21 (11.9)	8 (22.2)	47 (15.1)
Small insertion	1 (1.0)	1 (0.6)	3 (8.3)	5 (1.6)
Small deletion	5 (5.1)	7 (4.0)	1 (2.8)	13 (4.2)
Others	2 (2.0)	1 (0.6)	0	3 (1.0)

DMD, Duchenne muscular dystrophy.

### Time to life-altering clinical milestones due to DMD progression

**Fig 2:**
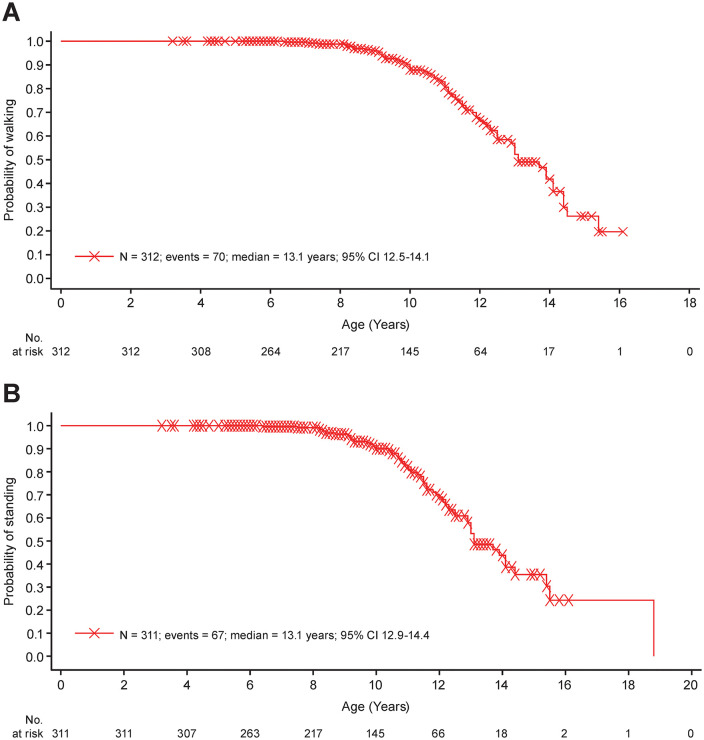
Time to life-altering clinical milestones due to DMD progression. **(A)** Age at failure to walk. **(B)** Age at failure to stand. Data are shown for the total study population. One participant with an event of failure to stand did not report failure age and was not included in the analysis.

The Kaplan–Meier estimate of the median (95% confidence interval [CI]) age at failure to walk was 13.1 (12.5–14.1) years in the total study population (Fig 2A). Median (95% CI) age at failure to walk was 14.4 (13.9-not evaluable) years in Group 2 and 11.1 (10.0–11.4) years in Group 3. No event of failure to walk was reported in Group 1. The median (95% CI) age at failure to stand was 13.1 (12.9–14.4) years in the total study population (Fig 2B), 14.4 (13.9-not evaluable) years in Group 2, and 11.0 (10.5–11.5) years in Group 3. No event of failure to stand was reported in Group 1. Only 2 events of failure to self-feed were reported, both in Group 3. Across groups, the median (95% CI) age at failure to self-feed was 20.0 (17.0-not evaluable) years.

### Motor function

**Fig 3:**
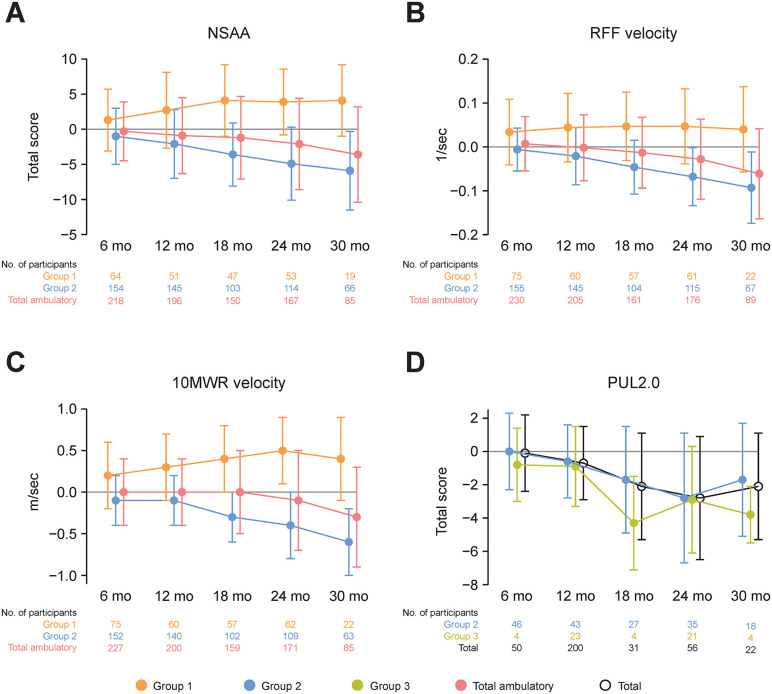
Change from baseline in motor function assessments up to 30 months. Data are presented as mean ± SD. Number of participants in each group for each motor function assessment is indicated. NSAA was only performed in ambulatory participants ≥3 years old. RFF velocity and 10MWR velocity were captured as part of the NSAA. PUL2.0 was only performed in participants ≥10 years old. For PUL2.0, only data with the same preferred arm as baseline were included for analysis. Invalid assessments were not included, based on third-party evaluation to validate whether the assessment had been successfully performed and qualified for inclusion. 10MWR, 10-meter walk/run; NSAA, North Star Ambulatory Assessment; PUL2.0, Performance of Upper Limb 2.0; RFF, rise from floor velocity.

Baseline values for motor function assessments are shown in S1 Table in S1 File. There was an improvement in NSAA in Group 1 but a decline in Group 2 and in the total ambulatory population (Fig 3A). The mean (SD) change from baseline to month 24 in NSAA total score was 3.9 (4.7) in Group 1, −4.9 (5.2) in Group 2, and −2.1 (6.5) in the total ambulatory population. Minimal changes were observed in RFF velocity in Group 1 but there were consistent declines in Group 2 and the total ambulatory population (Fig 3B). The mean (SD) change from baseline to month 24 for RFF was 0.047 (0.086) in Group 1, −0.068 (0.066) in Group 2, and −0.028 (0.091) in the total ambulatory population. Scores for 10MWR velocity did not decline in Group 1 but declined over time in Group 2, with no overall change in the total ambulatory population (Fig 3C). The mean (SD) change from baseline to month 24 in 10MWR was 0.5 (0.4) in Group 1, −0.4 (0.4) in Group 2, and −0.1 (0.6) in the total ambulatory population. Changes in PUL2.0 total score worsened over time and were similar in Groups 2 and 3 (Fig 3D).

### Muscle strength

Baseline values for muscle strength and changes from baseline at ≥2 years are shown in S1 Table in S1 File and S2 Fig, respectively. In general, decline in muscle strength was minimal across the groups, but loss of muscle strength appeared to be greater in the lower than the upper limbs. Although variable, deterioration was generally greater in the nonambulatory than the ambulatory participants.

### Range of motion

Baseline values for range of motion and changes from baseline at ≥2 years are shown in S1 Table in S1 File and S3 Fig, respectively. Range of motion in the ankles was lost across all groups, with the smallest losses in Group 1 and larger losses in Groups 2 and 3. There was little loss of range of motion in the elbow in Group 1, but loss of range of motion was much greater in Group 3. In general, loss of lower and upper limb range of motion was greater in nonambulatory than ambulatory participants.

### Pulmonary function

**Fig 4:**
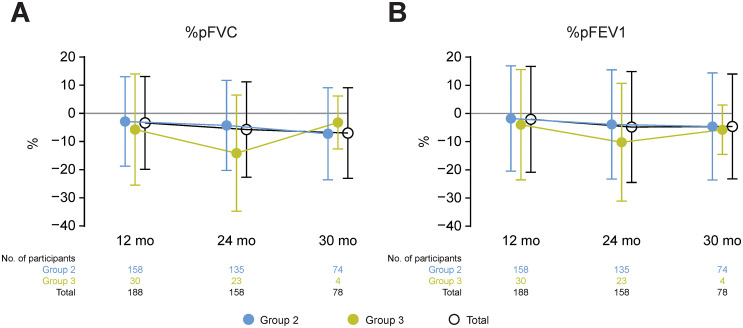
Change from baseline in %pFVC and %pFEV1 up to 30 months. Data are presented as mean ± SD. Number of participants in each group for each measure are indicated. Pulmonary function assessments were only carried out in participants ≥6 years old. Assessments were not carried out at 6 or 18 months. %pFEV1, percent predicted forced expiratory volume in 1 second; %pFVC, percent predicted forced vital capacity.

Pulmonary function assessments were conducted in participants ≥6 years old; therefore, data were available for Groups 2 and 3 only. Baseline data values are shown in S1 Table in S1 File. At ≥2 years, %pFVC decreased in both Group 2 and Group 3 compared with baseline, but to a greater extent in Group 3 (Fig 4A). Similarly, %pFEV1 deteriorated in both groups, but the deterioration was greater in Group 3 than in Group 2 (Fig 4B). Changes from baseline in MIP and MEP were minimal in both groups at all timepoints, except for at 30 months in Group 3 (S4A and B Fig), albeit with a small sample size in each case (n = 4). Peak cough flow increased in both groups, but to a greater extent in Group 3 (S4C Fig), albeit with large variability.

### LVEF

LVEF was assessed in participants ≥6 years old; therefore, data were available for Groups 2 and 3 only. Baseline values are shown in S2 Table in S1 File. Changes from baseline in LVEF were minimal in Groups 2 and 3, and in general at all timepoints (S2 Table in S1 File).

### WISC

The WISC assessment was conducted in ambulatory subjects ≥6 to ≤16 years old; therefore, data were only available for Group 2. At baseline, the mean (SD) full scale composite score for Group 2 was 85.0 (15.9). At 2 years, the mean (SD) change from baseline was 1.0 (8.6). No other timepoints were assessed.

### Quality of life and health care utilization endpoints

Baseline values for all versions of the PODCI Global Function Scale are shown in S3–5 Tables in S1 File. At baseline, mean (SD) standardized scores for Global Function using the pediatric parent report in participants ≤10 years old were 86.3 (8.6), 79.4 (12.7), 82.1 (11.8), 51.1 (7.6), and 81.0 (13.0) in Group 1, Group 2, total ambulatory participants, Group 3, and the total study population, respectively. Mean (SD) standardized scores for Global Function at baseline using the adolescent parent report in participants 11–18 years old were 69.5 (12.7), 42.5 (9.3), and 56.9 (17.6) in Group 2, Group 3, and the total study population. By comparison, scores at baseline in the same groups were numerically higher using the adolescent self-report: 76.4 (9.0), 50.3 (11.0), and 64.3 (16.4), respectively. Changes from baseline in PODCI Global Function at ≥2 years are also shown in S3–S5 Tables in S1 File. There was a consistent deterioration in Global Function scores across groups for each version. In general, there was also a consistent deterioration in each individual measure across groups and across versions. The largest deteriorations were seen in Group 2 using the adolescent parent report. Greater deterioration was observed in Groups 2 and 3 compared with Group 1.

Baseline values for EQ-5D-Y index and VAS scores are shown in S6 Table in S1 File. At ≥2 years, there was no change in EQ-5D-Y index score in either Group 1 or Group 3, but there was a deterioration in Group 2 and in the total population (S6 Table in S1 File). Changes from baseline in EQ-5D-Y VAS were largest in Groups 2 and 3. Scores at baseline and at ≥2 years for each EQ-5D-Y dimension are shown in S7 Table in S1 File. EQ-5D-3L was conducted in participants ≥16 years old, a total of four participants, all in Group 3. Because of this small sample size, data on EQ-5D-3L are not shown.

WPAI:CG scores at baseline and the change from baseline at ≥2 years are shown in S8 Table in S1 File. Scores at baseline for each measure of the WPAI:CG were consistently higher in Group 3 than in Groups 1 and 2. At ≥2 years, the changes across groups were minimal and inconsistent, including in Group 3.

## Discussion

These results extend the 1-year findings from the same study [25] up to a maximum of 30 months, albeit there was variability of data both within and among groups across the duration of the study so that these results should be interpreted with caution. The age at failure to walk reported with this extended follow-up is longer than the loss of ambulation of 10.4 years reported in another Chinese population, probably due to lower glucocorticoid use in the other study [22]. However, values were similar to those reported in natural history studies in other ethnic populations receiving glucocorticoids (e.g., loss of ambulation at 13.4 years) [2,18], and in a recent systematic review (range 9.5–12.5 years) [16]. It should be noted, however, that definitions for loss of ambulation and loss of failure to walk are not necessarily consistent across studies. For some measures, there was a clear deterioration between the 1-year and ≥2-year follow-ups, depending on cohort. For instance, NSAA total score deteriorated in Group 2, whereas it was stable in Group 1. NSAA scores improve in participants with DMD up to 6–7 years of age, after which they plateau and begin to decline [26]. Participants in Group 1 were all < 6 years of age whereas Group 2 included participants aged ≥6 years and for whom an improvement or plateau in motor function is not expected. The declines in timed motor tests described are similar to those reported previously over similar duration of follow-up [27,28]. For instance, our data showed a change of −5.0 points in NSAA at 2 years in Group 2, which compares with a change of −4.2 points in children aged >7 years followed for 24 months [27] and −9.5 points at 36 months [28]. These previous studies also mirrored our observations that deteriorations in NSAA were smaller or negligible in younger individuals [27,28]. There were minimal changes in RFF in Group 1 but consistent declines in Group 2 and the total ambulatory population. These were mirrored by worsening of lower limb muscle strength (knee extension) and joint range of motion (ankle) in Groups 2 and 3, but also in Group 1 up to 30 months (albeit with a small sample size at this timepoint). Upper limb motor function, as assessed by PUL2.0, deteriorated in Groups 2 and 3 up to 30 months, further reflecting a general loss of motor function across limbs. The changes observed in Groups 2 (−2.8 points) and 3 (−2.9 points) at 24 months were similar to those reported previously also after 24 months (−2.9 points) [29]. Assessments of arm muscle strength and joint range of motion in Group 1 in general indicated no deterioration up to 30 months. Changes over time in upper arm muscle strength and joint range of motion were more varied in Groups 2 and 3, but in general scores were worse than in Group 1.

Pulmonary function deteriorated from years 1 to 2 in Groups 2 and 3. The extent of the deteriorations in %pFVC at 2 years (−4.3% in Group 2 and −14.1% in Group 3) are similar to those reported previously in individuals receiving glucocorticoid treatment (range, −1.5% to −11.3% in those aged 7.0–14.9 years) [30]. Worsening of pulmonary function may also have occurred up to 30 months, but sample sizes were often small beyond 2 years, so these results should be interpreted with caution. Changes in LVEF in Groups 2 and 3 were minimal from years 1 to 2, and even up to 30 months, indicating stable cardiac function during this follow-up. Cardiac dysfunction usually becomes clinically apparent from the age of 10 years [31]. The mean ages of participants in Groups 2 and 3 were 9.0 and 12.7 years, respectively, therefore deteriorations in LVEF may have become apparent with longer follow-up. Again, the issue of small sample sizes for some cohorts at some timepoints needs to be considered when interpreting these data.

The 1-year data from this study [25] briefly described quality of life (QoL) findings in the form of the EQ-5D assessment. The EQ-5D data at ≥2 years reported here showed little change from 1 year in index scores for any group, although VAS scores did decline in Groups 2 and 3, indicating a reduction in QoL. This publication is the first time that PODCI and WPAI:CG have been reported for this study. For the PODCI, Global Function scores showed consistent decline across all groups for each version used. Moreover, there were also consistent declines in each of the individual subscales across groups and versions. Notably, the largest deteriorations were observed in Groups 2 and 3, highlighting the decline in QoL as DMD progresses. A previous study identified a decline in PODCI scores in a small population of subjects with DMD of similar age (mean, 7.9 years at baseline) at 1-year follow-up [32]. Our data extend these findings over a longer time course in a larger sample population. Changes in WPAI:CG up to 30 months were minimal in any group. To the best of our knowledge, this is the first time that the WPAI:CG has been used in a natural history study in DMD. The lack of change up to 30 months of follow-up suggests the WPAI:CG is either not sensitive enough to detect changes in caregiver work productivity over that time, or that a longer follow-up is required.

This publication is also the first time that the *DMD* mutations in this population have been reported. Exon deletion was the most common type of mutation followed by point mutation and exon duplication. These proportions are similar to those seen in other Chinese populations [20,21,33–37], although lower rates of exon deletions have also been reported [23]. Our findings are also similar to those reported in other ethnic populations [1,38–40]. In the current study, the greatest number of mutations occurred between exons 44 and 54, with an additional hotspot between exons 3 and 11. This is similar to previous reports in Chinese populations and other ethnic groups [36,37,39,41].

This study had several limitations, some of which have been reported previously [25]. In addition to those limitations, the study took place during the COVID-19 pandemic when visits to healthcare professionals were limited; therefore, some data points could not be captured for some participants. In particular, the number of visits to health care professionals was included as an endpoint in the study, but these data have not been reported. Not all participants were required to be assessed at Month 30, therefore sample sizes for some measures were small. This may affect the interpretation of the trajectory of some of the assessments in this study. Moreover, since DMD is a long-term disease, the maximum follow-up of 30 months reported here is not sufficiently long enough to fully understand disease progression. Finally, as discussed previously [25], no statistical comparisons were made, therefore the data should be interpreted with caution.

In conclusion, these results from a natural history study demonstrate the progression of DMD in Chinese boys of differing age and ambulatory status for up to 30 months follow-up. Natural history studies in diverse populations are required to fully understand the effects of race/ethnicity on DMD disease progression [42], and to enable the development of appropriate and effective treatments. Long-term natural history studies are also required to understand the time course of the disease more fully and to develop more sensitive assessments for DMD.

## Supporting information

S1 Fig Participant disposition at final follow-up. (TIFF)

S2 Fig Change from baseline in muscle strength up to 30 minutes. Data are presented as mean ± SD. Number of participants in each group for each measure are indicated. Muscle strength tests were only performed in participants ≥5 years old. Invalid assessments were not included in the analysis, based on third-party evaluation to validate whether the assessment had been successfully performed and qualified for inclusion.
(TIFF)

S3 Fig Change from baseline in joint range of motion up to 30 months. Data are presented as mean ± SD. Number of participants in each group for each measure are indicated.
(TIFF)

S4 Fig Change from baseline in MIP, MEP, and peak cough flow at 2 years. Data are presented as mean ± SD. Number of participants in each group for each measure are indicated. Pulmonary function assessments were only carried out in participants ≥6 years old. Assessments were not carried out at 6 or 18 months. MEP, maximum expiratory pressure; MIP, maximum inspiratory pressure.
(TIFF)

S1 File Supporting Tables (S1 Table – S8 Table). (DOCX)
