# Supplementary figures and images for: A natural history study of Chinese individuals with Duchenne muscular dystrophy: Results from 2 years of follow-up and beyond
Source: PLoS One. 2026 Apr 2;21(4):e0345023. doi: 10.1371/journal.pone.0345023 (PMC13046110; doi:10.1371/journal.pone.0345023)

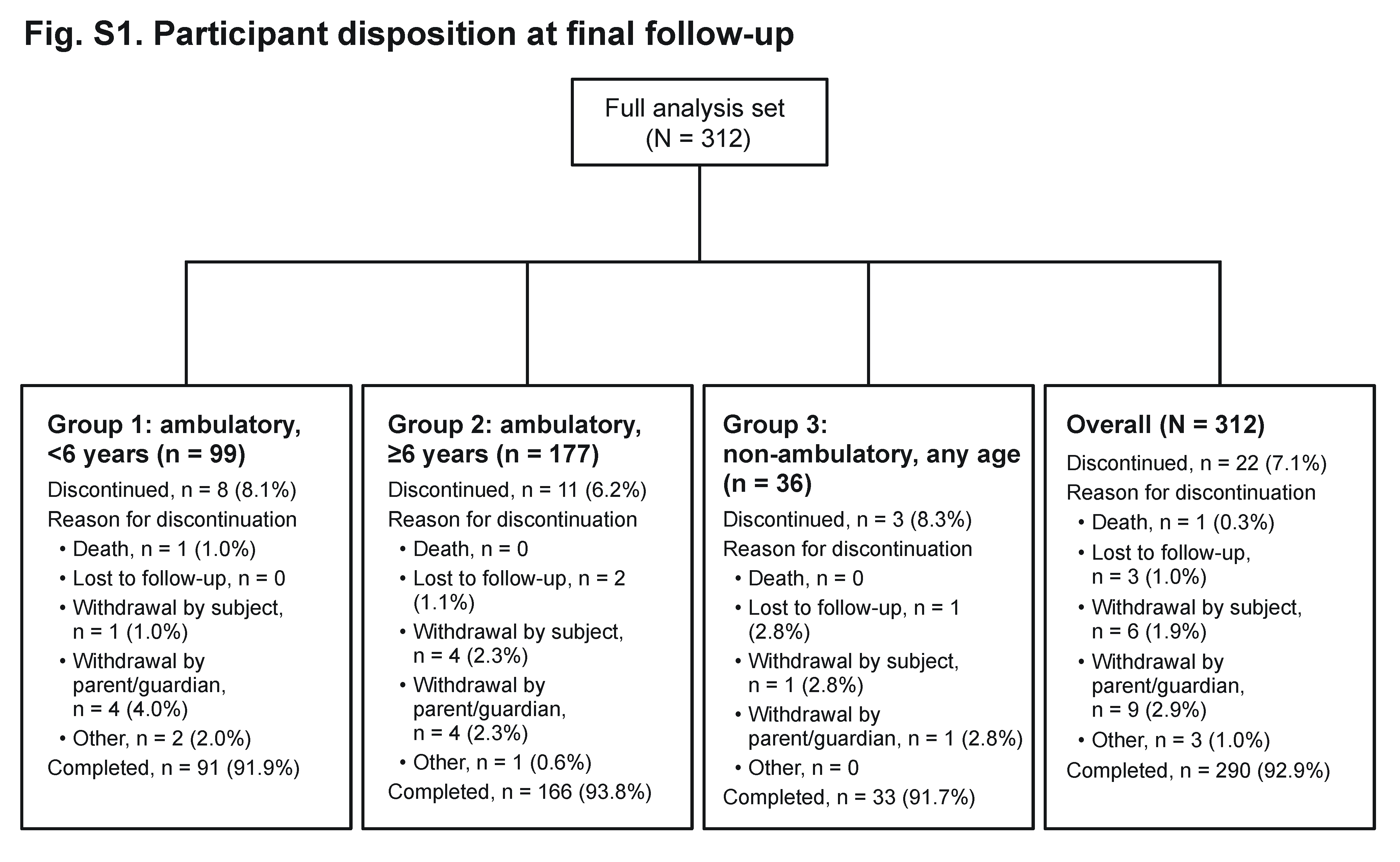

Supplement: S1 Fig — (TIFF) [file pone.0345023.s001.tiff]

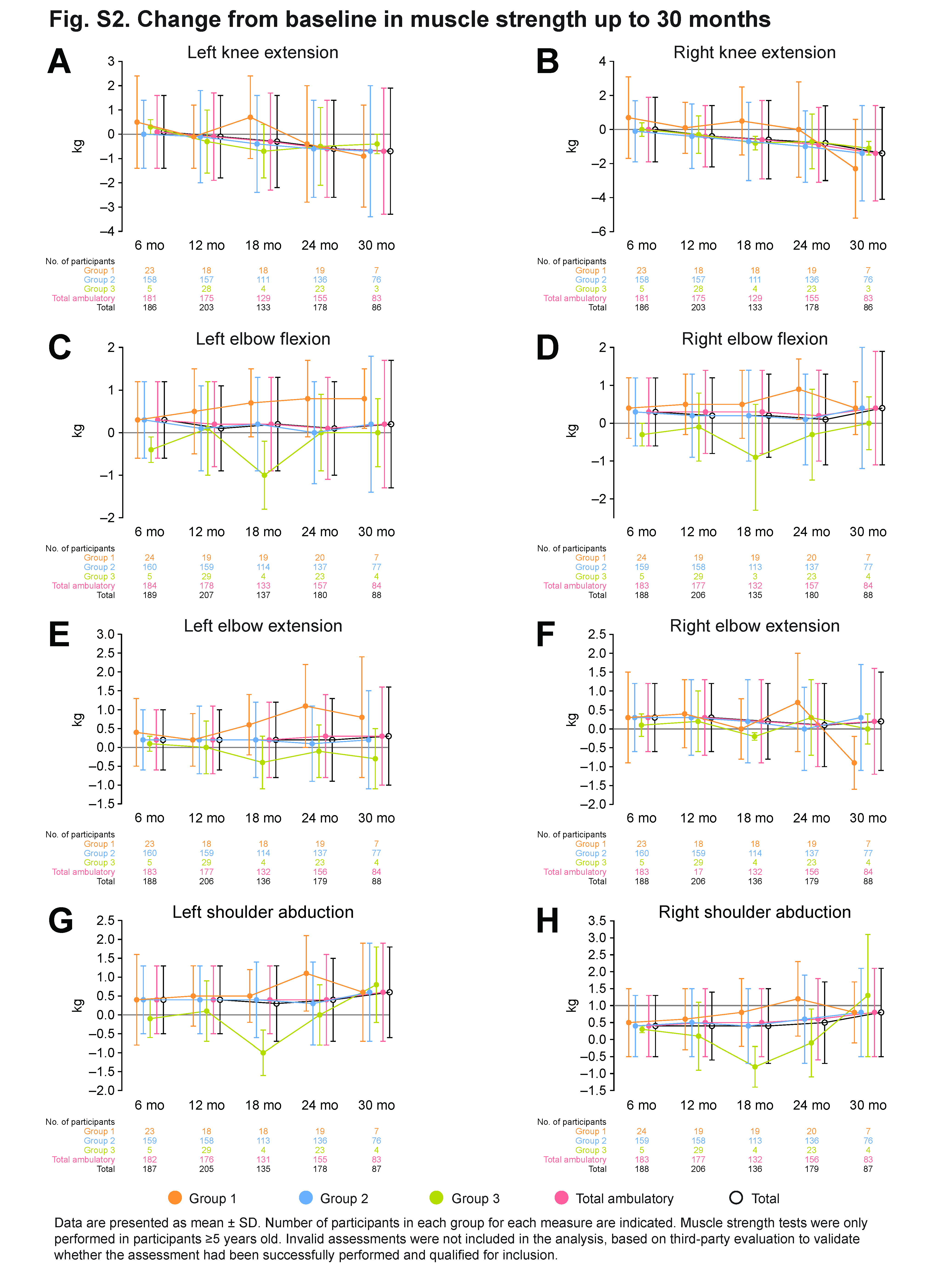

Supplement: S2 Fig — Data are presented as mean ± SD. Number of participants in each group for each measure are indicated. Muscle strength tests were only performed in participants ≥5 years old. Invalid assessments were not included in the analysis, based on third-party evaluation to validate whether the assessment had been successfully performed and qualified for inclusion. (TIFF) [file pone.0345023.s002.tiff]

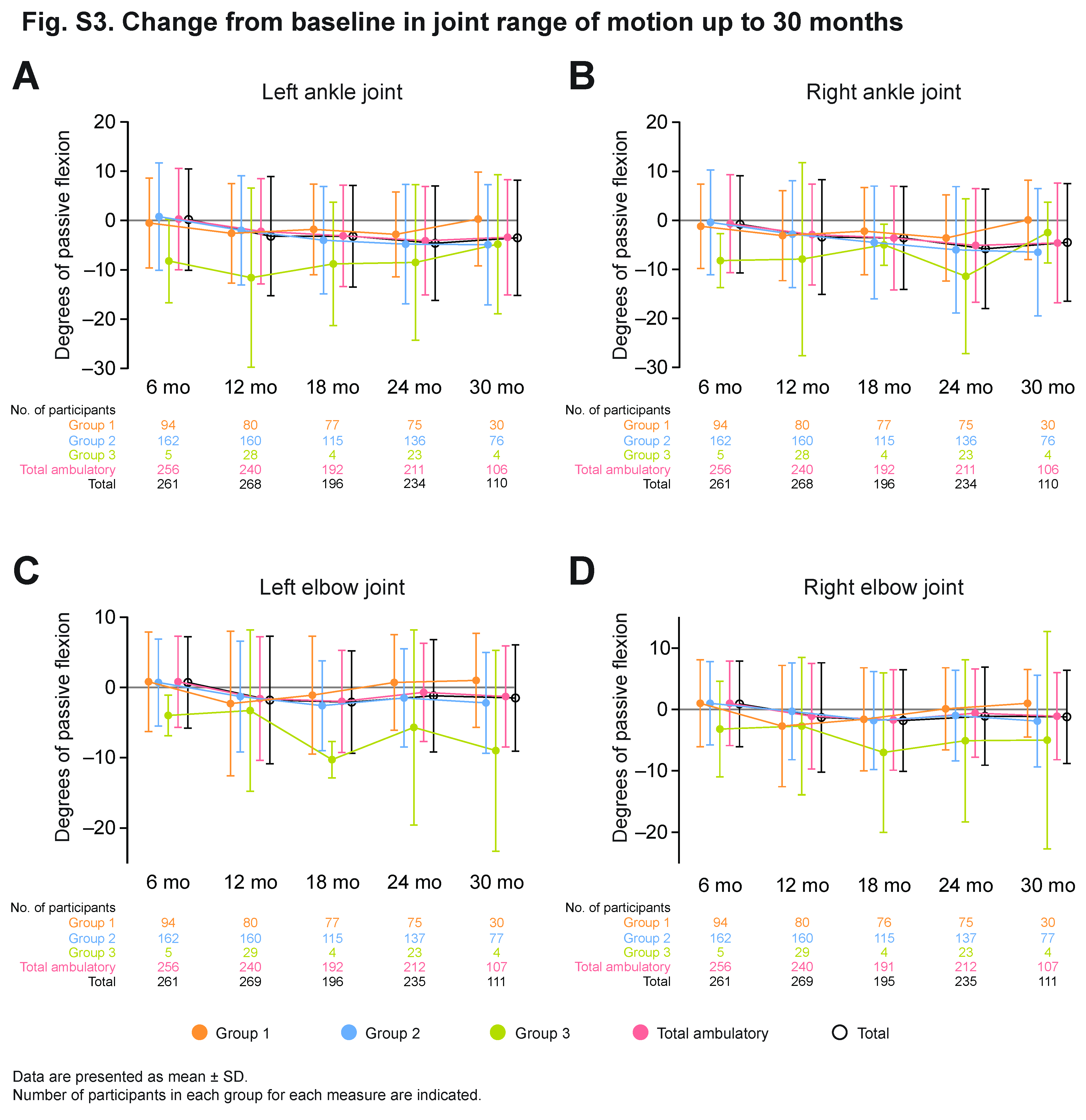

Supplement: S3 Fig — Data are presented as mean ± SD. Number of participants in each group for each measure are indicated. (TIFF) [file pone.0345023.s003.tiff]

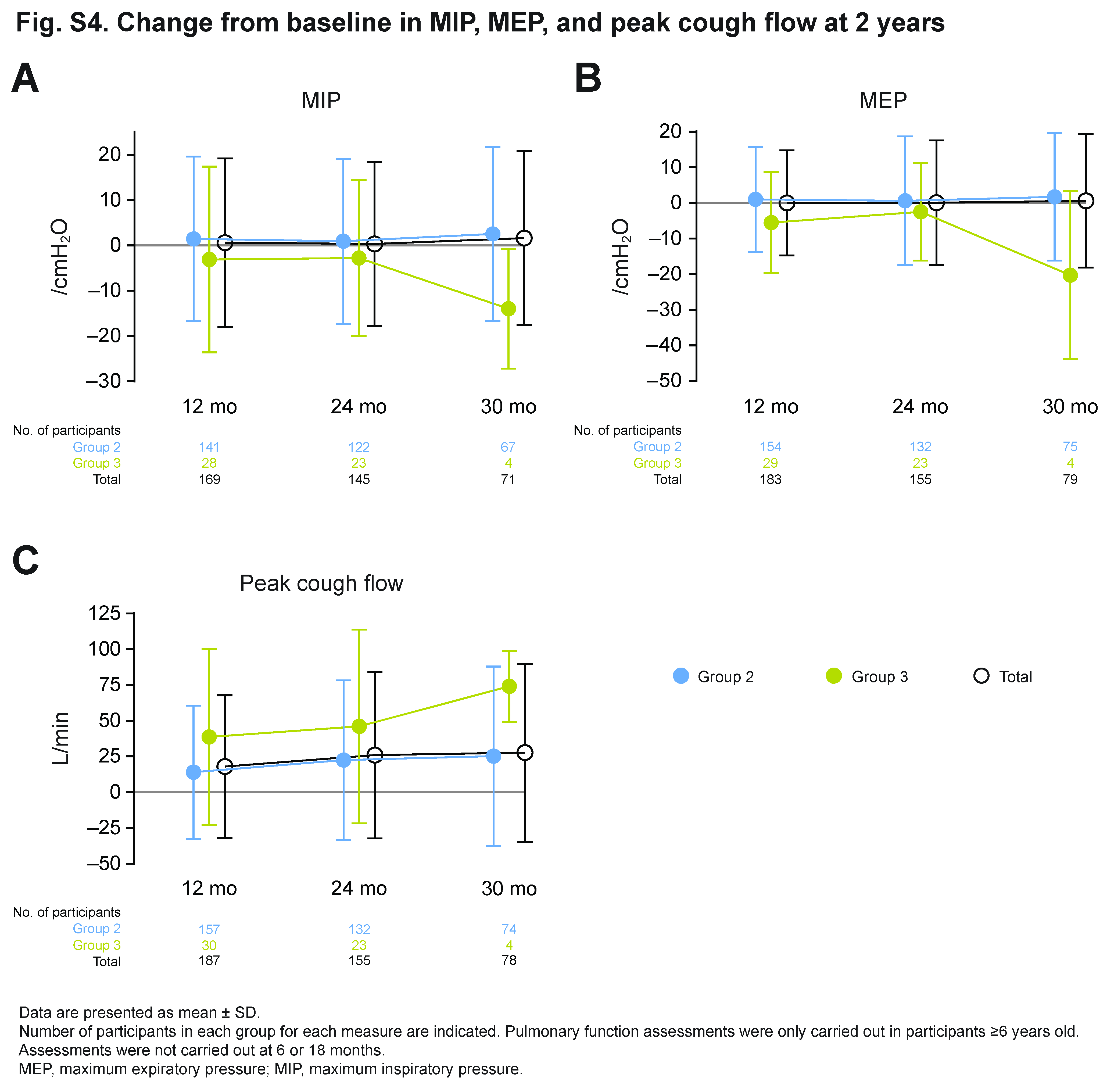

Supplement: S4 Fig — Data are presented as mean ± SD. Number of participants in each group for each measure are indicated. Pulmonary function assessments were only carried out in participants ≥6 years old. Assessments were not carried out at 6 or 18 months. MEP, maximum expiratory pressure; MIP, maximum inspiratory pressure. (TIFF) [file pone.0345023.s004.tiff]
