# Supplementary material for: A natural history study of Chinese individuals with Duchenne muscular dystrophy: Results from 2 years of follow-up and beyond
Source: PLoS One. 2026 Apr 2;21(4):e0345023. doi: 10.1371/journal.pone.0345023 (PMC13046110; doi:10.1371/journal.pone.0345023)
Supplement: S1 File — (DOCX) [file pone.0345023.s005.docx]

| **S1 Table. Baseline values for motor function, muscle strength, range of motion, pulmonary, LVEF, and EQ-5D assessments** | | | | | | | | | | |
| --- | --- | --- | --- | --- | --- | --- | --- | --- | --- | --- |
| **Assessment** | **Group 1:**  **ambulatory, age <6 y** | | **Group 2:**  **ambulatory, age ≥6 y** | | **Total ambulatory** | | **Group 3:**  **nonambulatory, any age** | | **Total** | |
|  | **n** | **Result** | **n** | **Result** | **n** | **Result** | **n** | **Result** | **N** | **Result** |
| **Motor function** | | | | | | | | | | |
| NSAA, total score | 68 | 21.4 (5.8) | 172 | 19.6 (8.3) | 240 | 20.1 (7.7) | – | – | – | – |
| RFF velocity, 1/sec | 78 | 0.199 (0.085) | 173 | 0.149 (0.117) | 251 | 0.165 (0.110) | – | – | – | – |
| 10MWR velocity, m/s | 78 | 1.9 (0.5) | 169 | 1.7 (0.6) | 247 | 1.7 (0.6) | – | – | – | – |
| PUL2.0, total score | – | – | 51 | 36.7 (4.9) | – | – | 29 | 26.8 (9.5) | 80 | 33.1 (8.4) |
| **Muscle strength, kg** | | | | | | | | | | |
| Left knee extension | 25 | 6.7 (2.7) | 174 | 4.2 (2.6) | 199 | 4.5 (2.7) | 35 | 1.8 (1.8) | 234 | 4.1 (2.8) |
| Right knee extension | 25 | 6.7 (2.6) | 174 | 4.4 (2.7) | 199 | 4.7 (2.8) | 35 | 1.9 (1.7) | 234 | 4.3 (2.9) |
| Left elbow flexion | 26 | 3.1 (0.9) | 176 | 3.5 (1.2) | 202 | 3.5 (1.1) | 36 | 2.6 (1.9) | 238 | 3.4 (1.3) |
| Right elbow flexion | 26 | 3.0 (0.9) | 175 | 3.4 (1.1) | 201 | 3.4 (1.0) | 36 | 2.6 (1.8) | 237 | 3.2 (1.2) |
| Left elbow extension | 25 | 3.0 (0.9) | 176 | 2.9 (1.1) | 201 | 2.9 (1.0) | 36 | 2.2 (1.3) | 237 | 2.8 (1.1) |
| Right elbow extension | 25 | 3.3 (1.2) | 176 | 3.2 (1.1) | 201 | 3.2 (1.1) | 36 | 2.2 (1.2) | 237 | 3.0 (1.2) |
| Left shoulder abduction | 25 | 2.6 (0.8) | 175 | 3.3 (1.1) | 200 | 3.2 (1.1) | 36 | 3.0 (1.6) | 236 | 3.1 (1.2) |
| Right shoulder abduction | 26 | 2.7 (0.9) | 175 | 3.3 (1.1) | 201 | 3.3 (1.1) | 36 | 3.1 (1.5) | 237 | 3.2 (1.1) |
| **Range of motion, degrees of passive flexion** | | | | | | | | | | |
| Left ankle | 99 | 13.3 (10.4) | 177 | 2.4 (10.8) | 276 | 6.3 (11.9) | 36 | −20.9 (20.3) | 312 | 3.2 (15.7) |
| Right ankle | 99 | 13.6 (10.4) | 177 | 2.1 (11.7) | 276 | 6.2 (12.5) | 36 | −22.4 (19.8) | 312 | 2.9 (16.3) |
| Left elbow | 99 | 9.9 (7.8) | 177 | 7.9 (7.7) | 276 | 8.6 (7.8) | 36 | −1.9 (14.7) | 312 | 7.4 (9.5) |
| Right elbow | 99 | 10.0 (7.7) | 177 | 7.4 (8.6) | 276 | 8.3 (8.4) | 36 | −2.7 (15.0) | 312 | 7.1 (10.0) |
| **Pulmonary** | | | | | | | | | | |
| %pFVC, % | – | – | 176 | 86.2 (17.1) | – | – | 36 | 68.2 (23.9) | 212 | 83.1 (19.6) |
| %pFEV1, % | – | – | 176 | 85.5 (18.4) | – | – | 36 | 65.4 (24.5) | 212 | 82.1 (20.9) |
| MIP, /cmH_2_O | – | – | 161 | 52.9 (20.4) | – | – | 34 | 61.9 (24.4) | 195 | 54.4 (21.3) |
| MEP, /cmH_2_O | – | – | 170 | 52.1 (17.7) | – | – | 35 | 52.9 (23.9) | 205 | 52.3 (18.9) |
| Peak cough flow, L/min | – | – | 175 | 187.1 (49.8) | – | – | 36 | 218.1 (72.6) | 211 | 192.4 (55.4) |
| Data are presented as mean (SD). NSAA was performed in ambulatory participants ≥3 years old. RFF velocity and 10MWR were captured as part of NSAA. PUL2.0 was performed in participants ≥10 years old. Muscle strength assessments were performed in participants ≥5 years old. Pulmonary functions assessments were performed in participants ≥6 years old.  %pFV1, percent predicted forced expiratory volume in 1 second; %pFVC, percent predicted forced vital capacity; 10MWR, 10-minute walk/run; EQ-5D, EuroQol 5 dimensions; LVEF, left ventricular ejection fraction; MEP, maximum expiratory pressure; MIP, maximum inspiratory pressure; NSAA, North Star Ambulatory Assessment; PUL2.0, Performance of Upper Limb 2.0; RFF, rise from floor velocity. | | | | | | | | | | |

| **S2 Table. LVEF at baseline and change from baseline up to 30 months** | | | | | | |
| --- | --- | --- | --- | --- | --- | --- |
| **Time point** | **Group 2:**  **ambulatory, age** ≥**6 y** | | **Group 3:**  **non-ambulatory, any age** | | **Total** | |
|  | **n** | **LVEF, %** | **n** | **LVEF, %** | **N** | **LVEF, %** |
| Baseline | 177 | 66.2 (4.4) | 36 | 63.1 (7.4) | 213 | 65.7 (5.1) |
| 6 mo | 4 | 1.5 (6.6) | 0 | – | 4 | 1.5 (6.6) |
| 12 mo | 159 | 0.5 (6.3) | 29 | -0.4 (6.3) | 188 | 0.4 (6.3) |
| 18 mo | 11 | 0.6 (5.9) | 0 | – | 11 | 0.6 (5.9) |
| 24 mo | 137 | −0.7 (5.4) | 24 | −1.6 (7.6) | 161 | −0.8 (5.8) |
| 30 mo | 78 | −0.3 (6.4) | 4 | −2.0 (4.7) | 82 | −0.4 (6.3) |
| Data are presented as mean (SD). LVEF was only assessed in participants ≥6 years old.  LVEF, left ventricular ejection fraction. | | | | | | |

| **S3 Table. PODCI Global Functioning Scale (pediatric parent report) scores at baseline and change from baseline up to 30 months** | | | | | | | | | | | |
| --- | --- | --- | --- | --- | --- | --- | --- | --- | --- | --- | --- |
|  | **Group 1:**  **ambulatory, age <6 y** | | **Group 2: ambulatory, age** ≥**6 y** | | **Total ambulatory** | | **Group 3: nonambulatory, any age** | | **Total** | | |
|  | **n** | **Standardized score** | **n** | **Standardized score** | **n** | **Standardized score** | **n** | **Standardized score** | | **N** | **Standardized score** |
| **Subscale** |  |  |  |  |  |  |  |  |  |  |  |
| **Upper extremity** | | | | | | | | | | | |
| Baseline | 94 | 90.0 (9.7) | 146 | 91.3 (8.3) | 240 | 90.8 (8.9) | 9 | 85.7 (9.8) | | 249 | 90.6 (8.9) |
| 12 mo | 75 | 0.7 (10.9) | 116 | −1.4 (11.1) | 191 | −0.6 (11.0) | 4 | −8.3 (17.6) | | 195 | −0.7 (11.2) |
| 24 mo | 72 | −0.8 (11.4) | 78 | −4.1 (11.8) | 150 | −2.5 (11.7) | 0 | – | | 150 | −2.5 (11.7) |
| 30 mo | 27 | 4.1 (9.9) | 33 | −2.2 (10.9) | 60 | 0.6 (10.9) | 0 | – | | 60 | 0.6 (10.9) |
| **Transfer and mobility** | | | | | | | | | | | |
| Baseline | 94 | 92.6 (7.9) | 146 | 87.4 (13.3) | 240 | 89.4 (11.8) | 9 | 31.1 (15.0) | | 249 | 87.3 (16.1) |
| 12 mo | 75 | 1.9 (7.7) | 116 | −5.5 (14.8) | 191 | −2.6 (13.0) | 4 | −2.8 (17.0) | | 195 | −2.6 (13.0) |
| 24 mo | 72 | −0.5 (8.7) | 78 | −9.8 (15.5) | 150 | −5.4 (13.5) | 0 | – | | 150 | −5.4 (13.5) |
| 30 mo | 27 | 1.0 (6.2) | 33 | −11.0 (17.8) | 60 | −5.6 (15.0) | 0 | – | | 60 | −5.6 (15.0) |
| **Sports** | | | | | | | | | | | |
| Baseline | 94 | 76.8 (13.5) | 146 | 62.4 (19.0) | 240 | 68.0 (18.4) | 9 | 9.7 (8.3) | | 249 | 65.9 (21.2) |
| 12 mo | 75 | 2.7 (14.8) | 116 | −7.5 (15.8) | 191 | −3.5 (16.1) | 4 | −4.3 (3.1) | | 195 | −3.5 (16.0) |
| 24 mo | 72 | −1.1 (14.6) | 78 | −12.4 (16.7) | 150 | −7.0 (16.7) | 0 | – | | 150 | −7.0 (16.7) |
| 30 mo | 27 | 1.1 (10.3) | 33 | −19.9 (20.5) | 60 | −10.5 (19.6) | 0 | – | | 60 | −10.5 (19.6) |
| **Pain** | | | | | | | | | | | |
| Baseline | 94 | 85.6 (14.7) | 146 | 76.2 (20.7) | 240 | 79.9 (19.1) | 9 | 77.4 (14.1) | | 249 | 79.8 (18.9) |
| 12 mo | 75 | −1.4 (16.7) | 116 | −5.0 (22.0) | 191 | −3.6 (20.1) | 4 | −6.8 (14.2) | | 195 | −3.7 (20.0) |
| 24 mo | 72 | −7.1 (18.5) | 78 | −8.5 (21.4) | 150 | −7.8 (20.0) | 0 | – | | 150 | −7.8 (20.0) |
| 30 mo | 27 | −6.4 (13.8) | 33 | −15.2 (19.3) | 60 | −11.2 (17.5) | 0 | – | | 60 | −11.2 (17.5) |
| **Happiness** | | | | | | | | | | | |
| Baseline | 94 | 82.6 (13.8) | 146 | 69.3 (21.1) | 240 | 74.5 (19.6) | 9 | 57.8 (22.4) | | 249 | 73.9 (19.9) |
| 12 mo | 75 | 2.4 (14.4) | 116 | −5.6 (23.5) | 191 | −2.4 (20.7) | 4 | −17.5 (11.9) | | 195 | −2.7 (20.7) |
| 24 mo | 72 | −4.4 (19.2) | 78 | −12.3 (21.4) | 150 | −8.5 (20.7) | 0 | – | | 150 | −8.5 (20.7) |
| 30 mo | 27 | 4.9 (12.7) | 33 | −12.8 (20.7) | 60 | −4.9 (19.5) | 0 | – | | 60 | −4.9 (19.5) |
| **Global function** | | | | | | | | | | | |
| Baseline | 94 | 86.3 (8.6) | 146 | 79.4 (12.7) | 240 | 82.1 (11.8) | 9 | 51.1 (7.6) | | 249 | 81.0 (13.0) |
| 12 mo | 75 | 1.0 (9.0) | 116 | −4.9 (12.1) | 191 | −2.6 (11.4) | 4 | −5.5 (6.4) | | 195 | −2.7 (11.3) |
| 24 mo | 72 | −2.3 (10.3) | 78 | −8.7 (11.0) | 150 | −5.7 (11.1) | 0 | – | | 150 | −5.7 (11.1) |
| 30 mo | 27 | −0.1 (5.8) | 33 | −12.0 (13.7) | 60 | −6.7 (12.4) | 0 | – | | 60 | −6.7 (12.4) |
| Data are presented as mean (SD). The pediatric parent report was completed by parents or caregivers of participants ≤10 years old.  PODCI, Pediatric Outcomes Data Collection Instrument. | | | | | | | | | | | |

| **S4 Table. PODCI Global Functioning Scale (adolescent parent report) scores at baseline and change from baseline up to 30 months** | | | | | | |
| --- | --- | --- | --- | --- | --- | --- |
|  | **Group 2:**  **ambulatory, ≥6 y** | | **Group 3:**  **non-ambulatory, any age** | | **Total** | |
| **Subscale** | **n** | **Standardized score** | **n** | **Standardized score** | **N** | **Standardized score** |
| **Upper extremity** | | | | | | |
| Baseline | 31 | 88.4 (10.6) | 27 | 72.6 (19.3) | 58 | 81.1 (17.1) |
| 12 mo | 25 | −4.4 (9.2) | 19 | −2.4 (16.2) | 44 | −3.5 (12.6) |
| 24 mo | 20 | −11.0 (13.5) | 17 | −7.5 (16.5) | 37 | −9.4 (14.8) |
| 30 mo | 7 | −7.4 (10.2) | 2 | −8.0 (11.3) | 9 | −7.6 (9.7) |
| **Transfer and mobility** | | | | | | |
| Baseline | 31 | 75.5 (16.3) | 27 | 24.6 (13.8) | 58 | 51.8 (29.7) |
| 12 mo | 25 | −14.6 (17.2) | 19 | −9.2 (13.3) | 44 | −12.2 (15.7) |
| 24 mo | 20 | −27.1 (21.8) | 17 | −10.3 (12.7) | 37 | −19.4 (19.8) |
| 30 mo | 7 | −28.4 (25.8) | 2 | −1.5 (6.4) | 9 | −22.4 (25.4) |
| **Sports** | | | | | | |
| Baseline | 31 | 43.8 (17.3) | 27 | 10.4 (9.9) | 58 | 28.3 (22.0) |
| 12 mo | 25 | −12.4 (15.4) | 19 | −5.4 (11.2) | 44 | −9.4 (14.1) |
| 24 mo | 20 | −23.8 (17.5) | 17 | −2.6 (9.0) | 37 | −14.1 (17.7) |
| 30 mo | 7 | −24.7 (17.7) | 2 | −9.5 (13.4) | 9 | −21.3 (17.4) |
| **Pain** | | | | | | |
| Baseline | 31 | 70.0 (18.3) | 27 | 62.1 (18.4) | 58 | 66.4 (18.6) |
| 12 mo | 25 | −9.0 (20.3) | 19 | 11.3 (35.0) | 44 | −0.3 (29.1) |
| 24 mo | 20 | −14.2 (20.4) | 17 | 4.9 (29.7) | 37 | −5.4 (26.5) |
| 30 mo | 7 | −25.9 (22.0) | 2 | 9.0 (12.7) | 9 | −18.1 (24.9) |
| **Happiness** | | | | | | |
| Baseline | 31 | 67.6 (20.9) | 27 | 58.3 (24.0) | 58 | 63.3 (22.7) |
| 12 mo | 25 | −17.4 (19.4) | 19 | 1.6 (22.2) | 44 | −9.2 (22.5) |
| 24 mo | 20 | −22.3 (20.2) | 17 | −2.1 (25.7) | 37 | −13.0 (24.8) |
| 30 mo | 7 | −17.9 (24.3) | 2 | −17.5 (24.8) | 9 | −17.8 (22.8) |
| **Global function** | | | | | | |
| Baseline | 31 | 69.5 (12.7) | 27 | 42.5 (9.3) | 58 | 56.9 (17.6) |
| 12 mo | 25 | −10.0 (9.3) | 19 | −1.5 (12.1) | 44 | −6.4 (11.3) |
| 24 mo | 20 | −19.0 (12.3) | 17 | −3.8 (12.6) | 37 | −12.0 (14.5) |
| 30 mo | 7 | −21.7 (16.2) | 2 | −2.5 (2.1) | 9 | −17.4 (16.4) |
| Data are presented as mean (SD). The adolescent parent report was completed by participants 11-18 years old and by their parents or caregivers, therefore data were not available in ambulatory participants <6 years of age at screening (Group 1).  PODCI, Pediatric Outcomes Data Collection Instrument. | | | | | | |

| **S5 Table. PODCI Global Functioning Scale (adolescent self-report) scores at baseline and change from baseline up to 30 months** | | | | | | |
| --- | --- | --- | --- | --- | --- | --- |
|  | **Group 2: ambulatory, ≥6 y** | | **Group 3: nonambulatory, any age** | | **Total** | |
| **Subscale** | **n** | **Standardized score** | **n** | **Standardized score** | **N** | **Standardized score** |
| **Upper extremity** | | | | | | |
| Baseline | 31 | 93.6 (4.7) | 27 | 81.1 (18.2) | 58 | 87.8 (14.2) |
| 12 mo | 25 | −4.6 (10.1) | 19 | −3.3 (13.5) | 44 | −4.0 (11.6) |
| 24 mo | 20 | −5.3 (10.9) | 17 | −5.5 (12.1) | 37 | −5.4 (11.3) |
| 30 mo | 7 | −5.4 (7.2) | 2 | −2.0 (2.8) | 9 | −4.7 (6.5) |
| **Transfer and mobility** | | | | | | |
| Baseline | 31 | 78.7 (13.0) | 27 | 29.6 (16.3) | 58 | 55.8 (28.7) |
| 12 mo | 25 | −10.6 (18.2) | 19 | −6.3 (16.4) | 44 | −8.8 (17.4) |
| 24 mo | 20 | −24.9 (19.6) | 17 | −7.9 (16.3) | 37 | −17.1 (19.9) |
| 30 mo | 7 | −17.0 (19.7) | 2 | −10.0 (8.5) | 9 | −15.4 (17.6) |
| **Sports** | | | | | | |
| Baseline | 31 | 49.0 (15.7) | 27 | 12.6 (10.8) | 58 | 32.1 (22.8) |
| 12 mo | 25 | −4.6 (19.6) | 19 | −3.2 (11.0) | 44 | −4.0 (16.3) |
| 24 mo | 20 | −21.4 (19.7) | 17 | −8.2 (12.6) | 37 | −15.3 (17.9) |
| 30 mo | 7 | −10.4 (13.5) | 2 | −1.5 (2.1) | 9 | −8.4 (12.3) |
| **Pain** | | | | | | |
| Baseline | 31 | 84.2 (17.2) | 27 | 78.0 (21.6) | 58 | 81.3 (19.5) |
| 12 mo | 25 | 2.1 (26.1) | 19 | 8.3 (20.5) | 44 | 4.8 (23.8) |
| 24 mo | 20 | −12.4 (24.2) | 17 | 4.0 (20.6) | 37 | −4.8 (23.8) |
| 30 mo | 7 | 13.9 (23.1) | 2 | 0 (0) | 9 | 10.8 (20.9) |
| **Happiness** | | | | | | |
| Baseline | 31 | 75.8 (21.8) | 27 | 72.3 (18.8) | 58 | 74.2 (20.4) |
| 12 mo | 25 | −4.8 (23.3) | 19 | 2.6 (15.6) | 44 | −1.6 (20.5) |
| 24 mo | 20 | −2.0 (31.5) | 17 | −10.6 (19.1) | 37 | −5.9 (26.5) |
| 30 mo | 7 | −2.9 (16.3) | 2 | −2.5 (10.6) | 9 | −2.8 (14.6) |
| **Global function** | | | | | | |
| Baseline | 31 | 76.4 (9.0) | 27 | 50.3 (11.0) | 58 | 64.3 (16.4) |
| 12 mo | 25 | −4.4 (12.7) | 19 | −0.9 (8.2) | 44 | −2.9 (11.0) |
| 24 mo | 20 | −15.9 (12.9) | 17 | −4.2 (7.4) | 37 | −10.5 (12.1) |
| 30 mo | 7 | −4.7 (12.5) | 2 | −3.5 (0.7) | 9 | −4.4 (10.8) |
| Data are presented as mean (SD). The adolescent self-report was completed by participants 11-18 years old only, therefore data were not available in ambulatory participants <6 years of age at screening (Group 1).  PODCI, Pediatric Outcomes Data Collection Instrument. | | | | | | |

| **S6 Table. EQ-5D-Y index and VAS scores at baseline and change from baseline up to 30 months** | | | | | | | | | | |
| --- | --- | --- | --- | --- | --- | --- | --- | --- | --- | --- |
|  | **Group 1:**  **ambulatory, age <6 y** | | **Group 2:**  **ambulatory, age ≥6 y** | | **Total ambulatory** | | **Group 3:**  **nonambulatory, any age** | | **Total** | |
| **Assessment** | **n** | **Score** | **n** | **Score** | **n** | **Score** | **n** | **Score** | **N** | **Score** |
| **EQ-5D-Y Index** | | | | | | | | | | |
| Baseline | 57 | 0.9 (0.1) | 175 | 0.9 (0.1) | 232 | 0.9 (0.1) | 32 | 0.6 (0.1) | 264 | 0.8 (0.2) |
| 12 mo | 45 | 0 (0.1) | 159 | 0 (0.2) | 204 | 0 (0.2) | 24 | 0 (0.1) | 228 | 0 (0.2) |
| 24 mo | 43 | 0 (0.1) | 133 | −0.1 (0.2) | 176 | −0.1 (0.2) | 15 | 0 (0.1) | 191 | −0.1 (0.2) |
| 30 mo | 18 | 0 (0.1) | 77 | −0.1 (0.2) | 95 | −0.1 (0.2) | 3 | 0 (0.1) | 98 | −0.1 (0.2) |
| **EQ-5D-Y VAS** | | | | | | | | | | |
| Baseline | 57 | 89.5 (12.2) | 175 | 84.3 (20.3) | 232 | 85.6 (18.7) | 32 | 86.0 (16.4) | 264 | 85.6 (18.4) |
| 12 mo | 45 | −1.0 (19.8) | 159 | −4.3 (23.0) | 204 | −3.5 (22.4) | 24 | −5.0 (22.9) | 228 | −3.7 (22.4) |
| 24 mo | 43 | −3.0 (15.1) | 133 | −5.6 (21.6) | 176 | −5.0 (20.2) | 15 | −5.9 (14.6) | 191 | −5.1 (19.8) |
| 30 mo | 18 | −1.0 (12.2) | 77 | −6.1 (21.7) | 95 | −5.1 (20.3) | 3 | 5.0 (5.0) | 98 | −4.8 (20.1) |
| Data are presented as mean (SD). EQ-5D-Y was only performed in participants <16 years old. Assessments were not carried out at 6 or 18 months.  EQ-5D-Y, EuroQoL 5 Dimension Youth; VAS, visual analog scale. | | | | | | | | | | |

| **S7 Table. EQ-5D-Y dimension scores at baseline and change from baseline up to 30 months** | | | | | | | | | | | | | | | | | | | | | |
| --- | --- | --- | --- | --- | --- | --- | --- | --- | --- | --- | --- | --- | --- | --- | --- | --- | --- | --- | --- | --- | --- |
|  | **Group 1: ambulatory, age <6 y** | | | | | **Group 2: ambulatory, age ≥6 y** | | | | **Total ambulatory** | | | | **Group 3: non-ambulatory, any age** | | | | **Total** | | | |
|  | **n** | **No problem** | **Som problem** | **Extreme problem** | **n** | | **No problem** | **Some problem** | **Extreme** | **n** | **No problem** | **Some problem** | **Extreme problem** | **n** | **No problem** | **Some problem** | **Extreme problem** | **N** | **No problem** | **Some problem** | **Extreme problem** |
| **Mobility, n (%)** | | | | | | | | | | | | | | | | | | | | | |
| Baseline | 57 | 48 (84.2) | 9 (15.8) | 0 | 175 | | 95 (54.3) | 75 (42.9) | 5 (2.9) | 232 | 143 (61.6) | 84 (36.2) | 5 (2.2) | 32 | 0 | 1 (3.1) | 31 (96.9) | 264 | 143 (54.2) | 85 (32.2) | 36 (13.6) |
| 12 mo | 57 | 45 (78.9) | 12 (21.1) | 0 | 160 | | 91 (56.9) | 52 (32.5) | 17 (10.6) | 217 | 136 (62.7) | 64 (29.5) | 17 (7.8) | 24 | 0 | 0 | 24 (100) | 241 | 136 (56.4) | 64 (26.6) | 41 (17.0) |
| 24 mo | 58 | 48 (82.8) | 10 (17.2) | 0 | 134 | | 56 (41.8) | 55 (41.0) | 23 (17.2) | 192 | 104 (54.2) | 65 (33.9) | 23 (12.0) | 15 | 0 | 0 | 15 (100) | 207 | 104 (50.2) | 65 (31.4) | 38 (18.4) |
| 30 mo | 26 | 26 (100) | 0 | 0 | 78 | | 34 (43.6) | 34 (43.6) | 10 (12.8) | 104 | 60 (57.7) | 34 (32.7) | 10 (9.6) | 3 | 0 | 0 | 3 (100) | 107 | 60 (56.1) | 34 (31.8) | 13 (12.1) |
| **Self-care, n (%)** | | | | | | | | | | | | | | | | | | | | | |
| Baseline | 57 | 18 (31.6) | 26 (45.6) | 13 (22.8) | 175 | | 99 (56.6) | 63 (36.0) | 13 (7.4) | 232 | 117 (50.4) | 89 (38.4) | 26 (11.2) | 32 | 3 (9.4) | 11 (34.4) | 18 (56.3) | 264 | 120 (45.5) | 100 (37.9) | 44 (16.7) |
| 12 mo | 57 | 22 (38.6) | 25 (43.9) | 10 (17.5) | 160 | | 70 (43.8) | 73 (45.6) | 17 (10.6) | 217 | 92 (42.4) | 98 (45.2) | 27 (12.4) | 24 | 3 (12.5) | 7 (29.2) | 14 (58.3) | 241 | 95 (39.4) | 105 (43.6) | 41 (17.0) |
| 24 mo | 58 | 32 (55.2) | 21 (36.2) | 5 (8.6) | 134 | | 65 (48.5) | 53 (39.6) | 16 (11.9) | 192 | 97 (50.5) | 74 (38.5) | 21 (10.9) | 15 | 1 (6.7) | 5 (33.3) | 9 (60.0) | 207 | 98 (47.3) | 79 (38.2) | 30 (14.5) |
| 30 mo | 26 | 16 (61.5) | 8 (30.8) | 2 (7.7) | 78 | | 45 (57.7) | 23 (29.5) | 10 (12.8) | 104 | 61 (58.7) | 31 (29.8) | 12 (11.5) | 3 | 0 | 2 (66.7) | 1 (33.3) | 107 | 61 (57.0) | 33 (30.8) | 13 (12.1) |

|  | **Group 1: ambulatory, age <6 y** | | | | **Group 2: ambulatory, age ≥6 y** | | | | **Total ambulatory** | | | | **Group 3: non-ambulatory, any age** | | | | **Total** | | | |
| --- | --- | --- | --- | --- | --- | --- | --- | --- | --- | --- | --- | --- | --- | --- | --- | --- | --- | --- | --- | --- |
|  | **n** | **No problem** | **Some problem** | **Extreme problem** | **n** | **No problem** | **Some problem** | **Extreme problem** | **n** | **No problem** | **Some problem** | **Extreme problem** | **n** | **No problem** | **Some problem** | **Extreme problem** | **n** | **No problem** | **Some problem** | **Extreme problem** |
| **Usual activities, n (%)** | | | | | | | | | | | | | | | | | | | | |
| Baseline | 57 | 41 (71.9) | 14 (24.6) | 2 (3.5) | 175 | 82 (46.9) | 87 (49.7) | 6 (3.4) | 232 | 123 (53.0) | 101 (43.5) | 8 (3.4) | 32 | 5 (15.6) | 16 (50.0) | 11 (34.4) | 264 | 128 (48.5) | 117 (44.3) | 19 (7.2) |
| 12 mo | 57 | 40 (70.2) | 17 (29.8) | 0 | 160 | 70 (43.8) | 74 (46.3) | 16 (10.0) | 217 | 110 (50.7) | 91 (41.9) | 16 (7.4) | 24 | 4 (16.7) | 6 (25.0) | 14 (58.3) | 241 | 114 (47.3) | 97 (40.2) | 30 (12.4) |
| 24 mo | 58 | 42 (72.4) | 15 (25.9) | 1 (1.7) | 134 | 49 (36.6) | 60 (44.8) | 25 (18.7) | 192 | 91 (47.4) | 75 (39.1) | 26 (13.5) | 15 | 6 (40.0) | 3 (20.0) | 6 (40.0) | 207 | 97 (46.9) | 78 (37.7) | 32 (15.5) |
| 30 mo | 26 | 16 (61.5) | 10 (38.5) | 0 | 78 | 28 (35.9) | 40 (51.3) | 10 (12.8) | 104 | 44 (42.3) | 50 (48.1) | 10 (9.6) | 3 | 3 (100) | 0 | 0 | 107 | 47 (43.9) | 50 (46.7) | 10 (9.3) |
| **Pain/discomfort, n (%)** | | | | | | | | | | | | | | | | | | | | |
| Baseline | 57 | 40 (70.2) | 17 (29.8) | 0 | 175 | 124 (70.9) | 49 (28.0) | 2 (1.1) | 232 | 164 (70.7) | 66 (28.4) | 2 (0.9) | 32 | 19 (59.4) | 11 (34.4) | 2 (6.3) | 264 | 183 (69.3) | 77 (29.2) | 4 (1.5) |
| 12 mo | 57 | 36 (63.2) | 21 (36.8) | 0 | 160 | 101 (63.1) | 57 (35.6) | 2 (1.3) | 217 | 137 (63.1) | 78 (35.9) | 2 (0.9) | 24 | 17 (70.8) | 7 (29.2) | 0 | 241 | 154 (63.9) | 85 (35.3) | 2 (0.8) |
| 24 mo | 58 | 38 (65.5) | 19 (32.8) | 1 (1.7) | 134 | 80 (59.7) | 52 (38.8) | 2 (1.5) | 192 | 118 (61.5) | 71 (37.0) | 3 (1.6) | 15 | 12 (80.0) | 3 (20.0) | 0 | 207 | 130 (62.8) | 74 (35.7) | 3 (1.4) |
| 30 mo | 26 | 17 (65.4) | 9 (34.6) | 0 | 78 | 44 (56.4) | 30 (38.5) | 4 (5.1) | 104 | 61 (58.7) | 39 (37.5) | 4 (3.8) | 3 | 3 (100) | 0 | 0 | 107 | 64 (59.8) | 39 (36.4) | 4 (3.7) |
| **Anxiety/depression, n (%)** | | | | | | | | | | | | | | | | | | | | |
| Baseline | 57 | 50 (87.7) | 7 (12.3) | 0 | 175 | 133 (76.0) | 33 (18.9) | 9 (5.1) | 232 | 183 (78.9) | 40 (17.2) | 9 (3.9) | 32 | 22 (68.8) | 10 (31.3) | 0 | 264 | 205 (77.7) | 50 (18.9) | 9 (3.4) |
| 12 mo | 57 | 45 (78.9) | 8 (14.0) | 4 (7.0) | 160 | 110 (68.8) | 41 (25.6) | 9 (5.6) | 217 | 155 (71.4) | 49 (22.6) | 13 (6.0) | 24 | 14 (58.3) | 9 (37.5) | 1 (4.2) | 241 | 169 (70.1) | 58 (24.1) | 14 (5.8) |
| 24 mo | 58 | 43 (74.1) | 14 (24.1) | 1 (1.7) | 134 | 91 (67.9) | 41 (30.6) | 2 (1.5) | 192 | 134 (69.8) | 55 (28.6) | 3 (1.6) | 15 | 11 (73.3) | 3 (20.0) | 1 (6.7) | 207 | 145 (70.0) | 58 (28.0) | 4 (1.9) |
| 30 mo | 26 | 18 (69.2) | 8 (30.8) | 0 | 78 | 48 (61.5) | 26 (33.3) | 4 (5.1) | 104 | 66 (63.5) | 34 (32.7) | 4 (3.8) | 3 | 1 (33.3) | 1 (33.3) | 1 (33.3) | 107 | 67 (62.6) | 35 (32.7) | 5 (4.7) |
| EQ-5D-Y was performed in participants <16 years old. Seven participants changed from EQ-5D-Y to EQ-5D-3L during the study. The number of participants (n) is the denominator for the percentages for each dimension. Assessments were not carried out at 6 or 18 months.  EQ-5D-3L, EuroQoL 5 Dimension 3 Level; EQ-5D-Y, EuroQoL 5 Dimension Youth. | | | | | | | | | | | | | | | | | | | | |

| **S8 Table. WPAI:CG scores at baseline and change from baseline up to 30 months** | | | | | | | | | | |
| --- | --- | --- | --- | --- | --- | --- | --- | --- | --- | --- |
|  | **Group 1:**  **ambulatory, age <6 y** | | **Group 2:**  **ambulatory, age ≥6 y** | | **Total ambulatory** | | **Group 3:**  **non-ambulatory, any age** | | **Total** | |
| **Measure, n (%)** | **n** | **Score** | **n** | **Score** | **n** | **Score** | **n** | **Score** | **N** | **Score** |
| **Work time missed** | | | | | | | | | | |
| Baseline | 71 | 10.3 (18.2) | 100 | 9.8 (19.4) | 171 | 10.0 (18.8) | 21 | 12.6 (23.6) | 192 | 10.2 (19.4) |
| 12 mo | 58 | 6.8 (15.0) | 89 | 7.3 (14.1) | 147 | 7.1 (14.4) | 16 | 21.1 (28.8) | 163 | 8.5 (16.8) |
| 24 mo | 60 | 10.5 (19.7) | 71 | 13.0 (23.1) | 131 | 11.9 (21.6) | 15 | 8.7 (16.5) | 146 | 11.5 (21.1) |
| 30 mo | 27 | 2.9 (6.7) | 40 | 13.8 (19.2) | 67 | 9.4 (16.2) | 3 | 0 (0) | 70 | 9.0 (16.0) |
| **Impairment while working** | | | | | | | | | | |
| Baseline | 71 | 33.8 (26.4) | 101 | 33.7 (26.7) | 172 | 33.7 (26.5) | 20 | 59.5 (27.6) | 192 | 36.4 (27.7) |
| 12 mo | 58 | 27.9 (23.9) | 89 | 41.3 (25.0) | 147 | 36.1 (25.4) | 15 | 40.7 (26.6) | 162 | 36.5 (25.4) |
| 24 mo | 59 | 32.5 (25.0) | 71 | 37.7 (25.9) | 130 | 35.4 (25.5) | 15 | 41.3 (28.0) | 145 | 36.0 (25.8) |
| 30 mo | 27 | 21.1 (24.2) | 40 | 45.3 (24.6) | 67 | 35.5 (27.0) | 3 | 56.7 (20.8) | 70 | 36.4 (27.0) |
| **Overall work impairment** | | | | | | | | | | |
| Baseline | 71 | 38.4 (29.0) | 99 | 38.4 (28.3) | 170 | 38.4 (28.5) | 20 | 62.4 (27.9) | 190 | 40.9 (29.3) |
| 12 mo | 58 | 32.0 (25.7) | 89 | 45.1 (26.0) | 147 | 39.9 (26.6) | 15 | 46.6 (29.4) | 162 | 40.5 (26.9) |
| 24 mo | 59 | 37.2 (27.6) | 70 | 44.3 (27.5) | 129 | 41.1 (27.7) | 15 | 45.9 (29.9) | 144 | 41.6 (27.9) |
| 30 mo | 27 | 23.3 (24.4) | 40 | 51.9 (25.5) | 67 | 40.4 (28.6) | 3 | 56.7 (20.8) | 70 | 41.1 (28.4) |
| **Activity impairment** | | | | | | | | | | |
| Baseline | 99 | 40.0 (30.1) | 177 | 44.6 (31.9) | 276 | 43.0 (31.3) | 36 | 66.9 (26.4) | 312 | 45.7 (31.7) |
| 12 mo | 80 | 30.9 (26.6) | 160 | 48.3 (29.0) | 240 | 42.5 (29.4) | 30 | 61.7 (31.6) | 270 | 44.6 (30.2) |
| 24 mo | 75 | 41.3 (29.4) | 137 | 51.8 (28.0) | 212 | 48.1 (28.9) | 23 | 62.6 (32.8) | 235 | 49.5 (29.5) |
| 30 mo | 30 | 23.7 (23.4) | 78 | 56.2 (26.5) | 108 | 47.1 (29.5) | 4 | 65.0 (31.1) | 112 | 47.8 (29.6) |
| Data are presented as mean (SD).  Data at 12, 24, and 30 months were collected from the same caregiver as at baseline. If a participant changed his caregiver during the reporting period, all values collected after the change were excluded. Assessments were not carried out at 6 or 18 months.  WPAI:CG, Work Productivity and Activity Impairment Questionnaire adapted for Caregivers. | | | | | | | | | | |
